# Identification of lung cancer drivers by comparison of the observed and the expected numbers of missense and nonsense mutations in individual human genes

**DOI:** 10.18632/oncotarget.28231

**Published:** 2022-05-25

**Authors:** Olga Y. Gorlova, Marek Kimmel, Spiridon Tsavachidis, Christopher I. Amos, Ivan P. Gorlov

**Affiliations:** ^1^Department of Medicine, Baylor College of Medicine, Houston, TX 77030, USA; ^2^Department of Statistics, Rice University, Houston, TX 77005, USA

**Keywords:** lung cancer, somatic mutations, driver genes, Catalog Of Somatic Mutations In Cancer (COSMIC)

## Abstract

Largely, cancer development is driven by acquisition and positive selection of somatic mutations that increase proliferation and survival of tumor cells. As a result, genes related to cancer development tend to have an excess of somatic mutations in them. An excess of missense and/or nonsense mutations in a gene is an indicator of its cancer relevance. To identify genes with an excess of potentially functional missense or nonsense mutations one needs to compare the observed and expected numbers of mutations in the gene. We estimated the expected numbers of missense and nonsense mutations in individual human genes using (i) the number of potential sites for missense and nonsense mutations in individual transcripts and (ii) histology-specific nucleotide context-dependent mutation rates. To estimate mutation rates defined as the number of mutations per site per tumor we used silent mutations reported in the Catalog Of Somatic Mutations In Cancer (COSMIC). The estimates were nucleotide context dependent. We have identified 26 genes with an excess of missense and/or nonsense mutations for lung adenocarcinoma, 18 genes for small cell lung cancer, and 26 genes for squamous cell carcinoma of the lung. These genes include known genes and novel lung cancer gene candidates.

## INTRODUCTION

The most common type of somatic mutations detected in tumor samples is single nucleotide substitutions (SNSs). SNSs in the coding regions lead to missense, silent, or nonsense mutations depending on the type of the substitution and the reading frame position. Size is a strong predictor of the number of SNSs in a gene [1, 2]. The number of mutations also depends on the nucleotide composition of the gene [3]. Mutation rates are context dependent, as they depend on adjacent nucleotides for the same type of nucleotide substitution [4–7]. Mutation rates also depend on the strand (sense/antisense) on which the initial mutational event takes place [8]. Gene features like gene expression level [9] or relative replication time influence its propensity to mutate [10]. It is possible to computationally mutate all nucleotide positions and, therefore, estimate the number of potential sites for missense, nonsense or silent mutations in individual transcripts. This can be done by taking into account the nucleotide context, i.e. the preceding and the subsequent nucleotides relative to a given site.

After missense mutations, silent mutations are the second most common type of somatic mutations produced by SNSs detected in tumor samples. The majority of silent mutations are expected to be neutral since they do not change the amino acid sequence, despite anecdotal examples of functionality (Bali & Bebok, 2015; Pagani, Raponi, & Baralle, 2005). Their selective neutrality makes silent mutations ideal for an unbiased assessment of mutation rate free of effects of selection. In this study, we used silent mutations to estimate nucleotide context-dependent mutation rates for different SNSs. We focused on lung cancer (LC) because it is the top cancer killer worldwide and because LC has one of the highest frequencies of somatic mutations [11–13].

Here we used silent mutations to estimate nucleotide context-dependent mutation rates that were defined as the number of somatic mutations per site per tumor. The expected numbers of missense and nonsense mutations in individual human transcripts were estimated based on the observed numbers of silent mutations and the number of potential sites weighted by the corresponding mutation rates. In other words, the expected numbers of missense and nonsense mutations were estimated under the assumption that they behave like silent (selectively neutral) mutations. A comparison of the observed and expected number of mutations identified genes with an excess of potentially functional missense and/or nonsense mutations. This was done separately for lung adenocarcinoma, squamous cell carcinoma and small cell lung cancer. We identified known as well as novel candidate cancer genes for lung cancer.

## RESULTS

### The number of missense and nonsense mutations per site per sample

**Figure 1:**
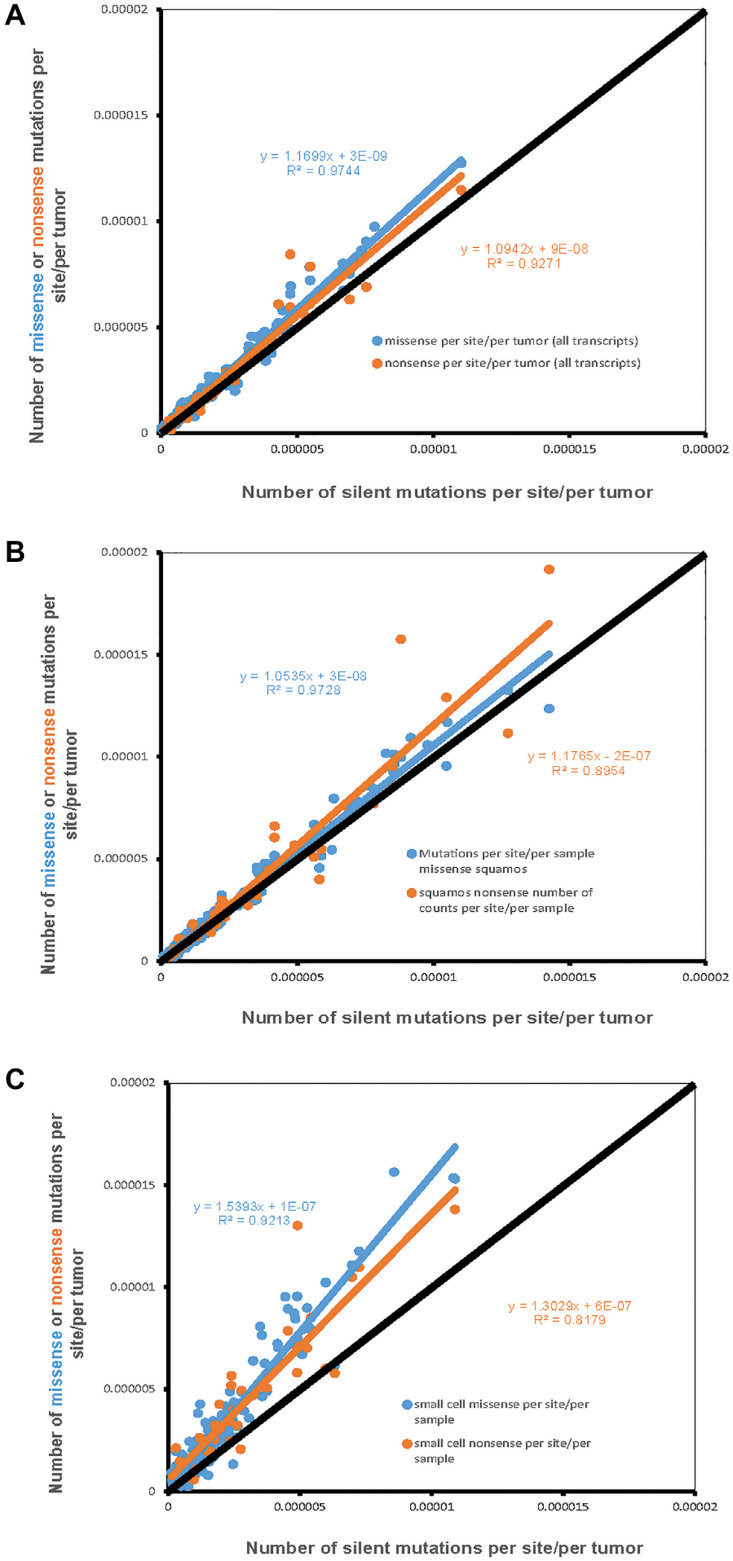
The number of missense and nonsense mutations per site per sample (Y-axis) compared to the number of silent mutations per site per sample (X-axis). Each dot represents one of 192 possible nucleotide context-dependent single nucleotide substitutions (NCD-SNS). Black line represents the expected number of mutations based on the number of silent mutations (per site per sample). (**A**) Adenocarcinoma, (**B**) Squamous cell carcinoma, (**C**) Small cell lung cancer.

For the 162 possible nucleotide context-dependent single nucleotide substitutions (NCD-SNSs) with available silent and missense mutations there was a strong positive correlation between the number of silent mutations per site per sample and the number of missense mutations per site per sample in all cell types (Figure 1). Pearson’s *ρ* was similar for adenocarcinoma (0.974, *N* = 162, *p* < 10^−16^) (Figure 1A) and squamous cell carcinoma (0.972, *N* = 162, *p* < 10^−16^) (Figure 1B). For small cell lung cancer, Pearson’s *ρ* was 0.921. We also observed a significant positive correlation between the number of silent mutations per site per sample and the number of nonsense mutations per site per sample (for small cell lung cancer, 0.818, *N* = 64, *p* = 10^−9^, and even higher for adenocarcinoma and squamous cell carcinoma).

The other important observation was that the number of missense and nonsense mutations per site per sample tended to be higher compared to the number of silent mutations per site per sample. The latter is evident from the slope of the linear regression lines for missense and nonsense mutations, placing them above the diagonal. This is further confirmed by the comparison of the mean differences between missense and silent and nonsense and silent mutations. For missense minus silent mutations, the corresponding *t*-tests for adenocarcinoma, squamous cell carcinoma and small cell carcinomas were: *t* = 7.7, *n* = 162, *p* = 1.5 × 10^−11^, *t* = 4.4, *n* = 162, *p* = 1.7 × 10^−5^, *t* = 11.5, *n* = 162, *p* = 1.5 × 10^−21^. The corresponding numbers for differences between the number of nonsense mutations per site and the number of silent mutations per site were: *t* = 2.8, *n* = 64, *p* = 0.008 for adenocarcinoma, *t* = 2.0, *n* = 64, *p* = 0.04 for squamous cell carcinoma, and *t* = 5.8, *n* = 64, *p* = 9.9 × 10^−8^ for small cell lung cancer.

### Mutation rates for 192 NCD-SNSs in adenocarcinoma, squamous cell carcinoma and small cell lung cancer

**Figure 2:**
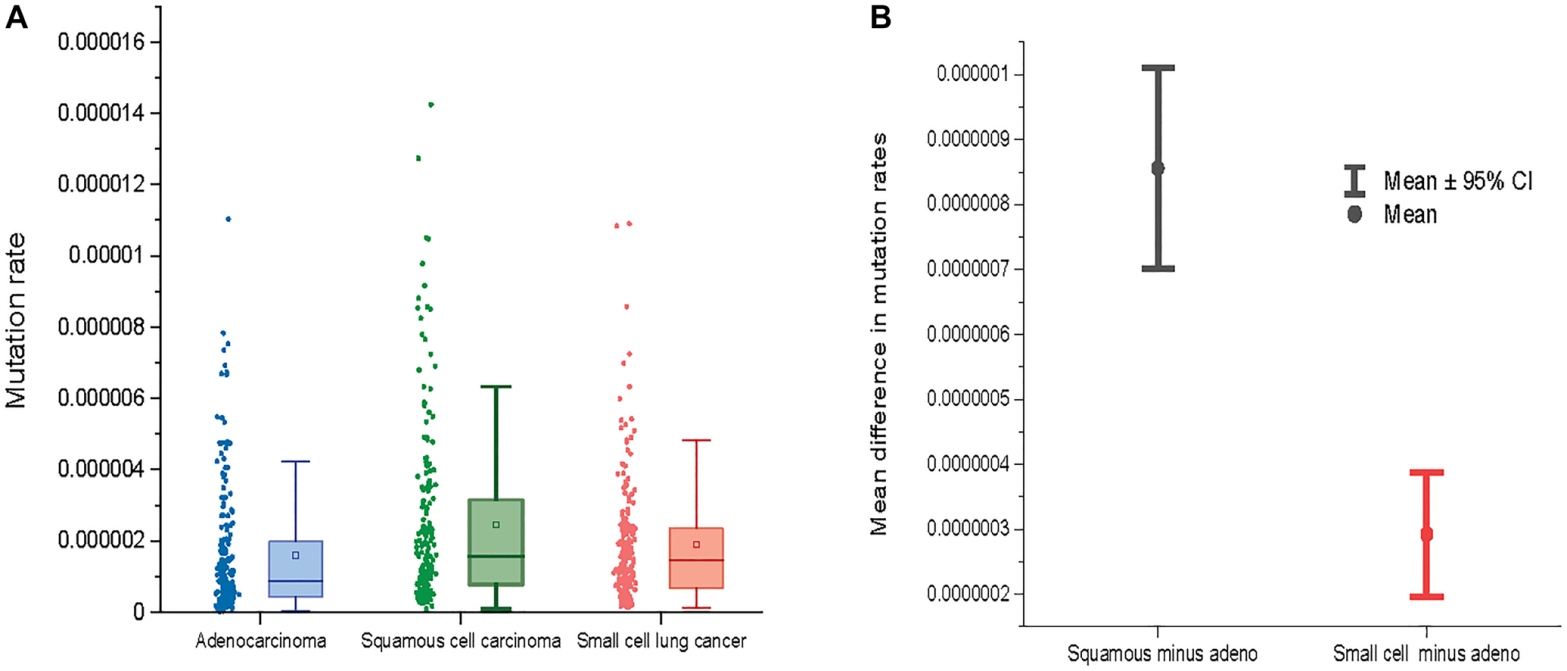
(**A**) Mutation rates across three major lung cancer cell types. (**B**) The mean difference between mutation rates for squamous cell lung cancer and adenocarcinoma (left) and between small cell lung cancer and adenocarcinoma (right) across 192 NCD-SNS.

Supplementary Figure 1 shows mutation rates for 192 NCD-SNSs in adenocarcinoma, squamos cell carcinoma and small cell lung cancer estimated using silent mutations only. There is more than 100× differences in mutation rates for different NCD-SNSs. When different histologies are compared, squamous and small cell lung cancer tend to have higher mutation rates than adenocarcinoma (Figure 2). The estimated mutation rates for 192 NCD-SNSs can be found in Supplementary Table 1.

Figure 2 shows a comparison of mutation rates across all the 192 NCD-SNS for the three lung cancer cell types. Lung adenocarcinoma has the lowest mutation rate while mutation rates for squamous and small cell lung cancer are higher (Figure 2A). The mutation rates for squamous cell lung cancer deviates from the mutation rates for adenocarcinoma stronger than does the mutation rate for small cell lung cancer, which is evident from the mean paired differences in mutation rates across the 192 NCD-SNS (Figure 2B).

### Accounting for mutation rates predicts the observed number of missense and nonsense mutations better than does the number of potential sites

**Figure 3:**
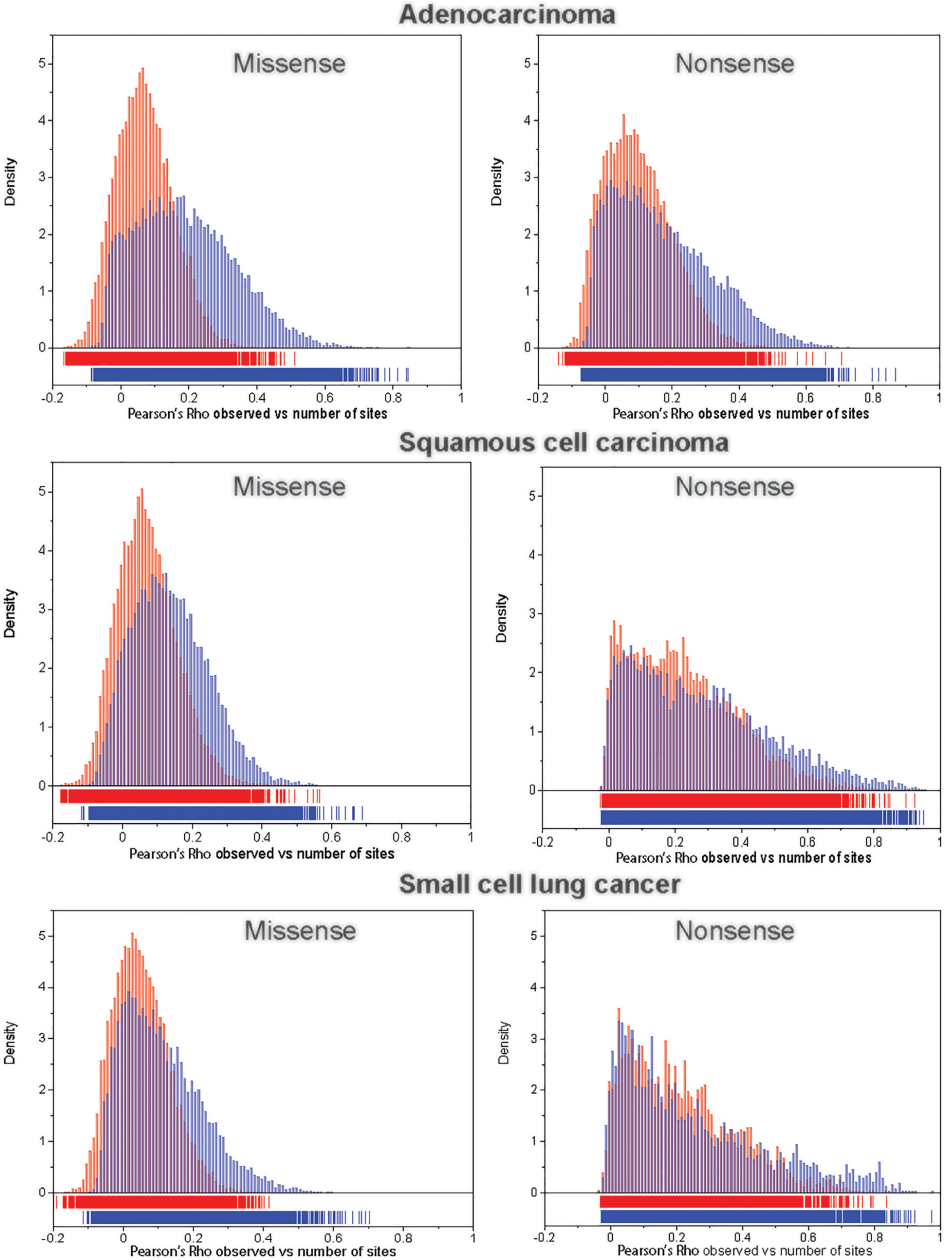
The distribution of Pearson’s correlation coefficient, ρ, between the observed number of mutations and the number of potential sites (red bars) and between the observed number of mutations and the number of potential sites weighted (multiplied) by the corresponding mutation rate (blue bars), for missense (left panel) and nonsense (right panel) mutations across 192 NCD-SNSs.

For each transcript we computed (1) the Pearson’s *ρ* between the observed number of missense and nonsense mutations and the number of corresponding potential sites across the 192 NCD-SNSs; and (2) the Pearson’s *ρ* between the observed number of missense and nonsense mutations and the number of potential sites weighted by the corresponding mutation rate as described in Materials and Methods section. Figure 3 shows the distributions of correlation coefficients for missense (left panels) and nonsense (right panels) mutations for the three LC histologies. We observed that the correlation coefficients were higher between the observed number of mutations and the number of potential sites weighted by the mutation rates of the corresponding NCD-SNS compared to the correlations between the observed number of mutations and the number of potential sites only.

### Genes with the higher than expected number of missense and/or nonsense mutations

Supplementary Figure 2 shows the distributions of the difference LOG(OBS/EXP)_mis or non_ - LOG(OBS/EXP)_silent_. As a threshold for identification of the genes associated with lung tumorigenesis we used LOG(OBS/EXP)_mis or non_ - LOG(OBS/EXP)_silent_ = 3. The threshold was selected because only a fraction of transcripts, less than 1%, exceeds it and because the majority of known lung cancer related genes exceed this threshold.

Using the threshold of 3 for the difference, 26 candidate genes for lung adenocarcinoma, 26 genes for squamous cell carcinoma and 18 for small cell carcinoma were identified. Table 1 shows the list of genes with the corresponding observed and expected numbers of missense and nonsense mutations and the silent mutation-corrected LOG ratio. Detailed information about candidate genes, additionally including those with the threshold of 2.5, can be found in Supplementary Table 2.

**Table 1:** List of candidate genes for adenocarcinoma, squamous cell carcinoma and small cell carcinoma identified by excess of missense or nonsense mutations

Histology	Gene	OBS missense	EXP missense	LOG(OBS/EXP)^*^	OBS nonsense	EXP nonsense	LOG(OBS/EXP)^*^
Adeno	TP53	145	1.88	5.62	24	0.16	5.14
Adeno	KRAS	165	1.34	5.14	0	0.14	
Adeno	ARID1A	14	19.2	2.46	13	1.43	4.56
Adeno	RBM10	12	7.89	1.35	17	0.71	3.71
Adeno	RB1	12	6.61	2.15	12	0.74	3.61
Adeno	STK11	28	4.12	1.43	26	0.30	3.60
Adeno	ENHO	1	0.7	0.27	1	0.03	3.42
Adeno	KRTAP19-2	1	0.43	0.68	2	0.06	3.36
Adeno	KRTAP19-4	2	0.67	0.96	3	0.09	3.32
Adeno	KRTAP6-2	10	0.57	3.25	0	0.07	
Adeno	BRAF	24	6.03	3.23	2	0.62	1.40
Adeno	CD109	25	10.2	3.22	2	1.02	1.39
Adeno	DEFB119	7	0.67	3.20	0	0.06	
Adeno	BICRA	8	15.6	0.77	4	0.70	3.18
Adeno	FXYD7	1	0.68	0.43	1	0.03	3.16
Adeno	ARID1B	23	21.41	3.16	3	1.63	2.55
Adeno	GNG13	6	0.55	3.14	0	0.06	
Adeno	RPS29	1	0.58	0.64	1	0.03	3.14
Adeno	HSPA12A	16	5.91	3.12	1	0.47	1.21
Adeno	URGCP-MRPS24	2	1.08	0.67	3	0.11	3.10
Adeno	DMXL1	27	21.7	3.09	4	2.19	2.08
Adeno	DACT1	19	7.71	3.08	0	0.60	
Adeno	ATP5F1E	0	0.35		1	0.04	3.04
Adeno	ADGRB1	18	15.8	3.04	1	1.05	1.52
Adeno	CHST9	5	0.52	3.03	0	0.06	
Adeno	SATB2	19	6.25	3.01	3	0.55	2.35
Squamous	CDKN2A	34	2.05	3.13	27	0.09	5.79
Squamous	TP53	233	1.97	5.13	33	0.16	4.59
Squamous	RASA1	12	10.05	2.20	9	0.87	3.67
Squamous	SPANXN1	9	0.65	3.60	1	0.08	2.07
Squamous	HLA-A	7	4.26	1.86	5	0.30	3.55
Squamous	DEFB110	4	0.56	2.72	2	0.06	3.46
Squamous	DEFB106A	1	0.55	0.90	1	0.03	3.43
Squamous	KCNN2	24	5.85	3.39	1	0.41	1.26
Squamous	SLC35G3	4	3.41	1.21	3	0.18	3.37
Squamous	NEFM	24	9.55	3.37	2	1.12	1.46
Squamous	LCE3D	4	1.05	1.89	2	0.06	3.35
Squamous	BICRA	3	18.11	1.11	2	0.77	3.31
Squamous	PASK	20	13.91	3.25	0	1.08	
Squamous	SEC61G	2	0.61	1.69	1	0.03	3.23
Squamous	KRTAP21-1	8	0.78	3.20	1	0.05	2.68
Squamous	TSHZ2	21	10.31	3.17	3	0.86	2.45
Squamous	CPAMD8	17	21.31	3.16	2	1.40	2.46
Squamous	ARID1A	14	22.9	1.72	8	1.60	3.09
Squamous	KLB	19	10.41	3.06	0	0.86	
Squamous	FOXI2	2	3.93	0.41	2	0.19	3.06
Squamous	CFH	29	11.51	3.06	6	1.33	2.41
Squamous	H3F3A	1	1.51	-0.40	2	0.08	3.05
Squamous	TMPRSS9	15	11.91	3.04	2	0.76	2.56
Squamous	ITGB4	16	20.62	3.02	0	1.31	
Squamous	SAGE1	24	8.72	3.02	2	0.62	1.81
Squamous	RPL39L	0	0.48		1	0.04	3.02
Small cell	TP53	106	0.51	6.36	19	0.04	4.74
Small cell	KIAA1211	31	3.14	3.92	1	0.29	0.39
Small cell	SPHKAP	34	3.69	3.88	1	0.31	0.37
Small cell	MUC12	26	12.05	3.77	1	0.82	1.05
Small cell	POLR2K	3	0.11	3.60	0	0.01	
Small cell	BLCAP	0	0.19		2	0.01	3.43
Small cell	KLK8	2	0.08	3.41	0	0.01	
Small cell	KRTAP23-1	3	0.13	3.33	0	0.01	
Small cell	FAM47C	22	2.62	3.33	1	0.19	0.52
Small cell	OTOF	19	5.07	3.30	1	0.35	0.84
Small cell	ZNF208	28	2.43	3.27	4	0.33	1.02
Small cell	NPAS3	17	2.47	3.19	0	0.16	
Small cell	MYO16	17	4.28	3.15	1	0.33	0.70
Small cell	CACNA1I	15	5.79	3.14	0	0.32	
Small cell	ALG10B	5	0.27	3.12	0	0.02	
Small cell	CD1E	5	0.28	3.05	1	0.02	1.71
Small cell	PCDHA3	14	2.38	3.03	1	0.16	0.88
Small cell	HMCN1	18	11.98	3.02	3	0.97	1.89

^*^The LOG ratios of the observed to the expected numbers of mutations are adjusted for the excess of silent mutations.

**Figure 4:**
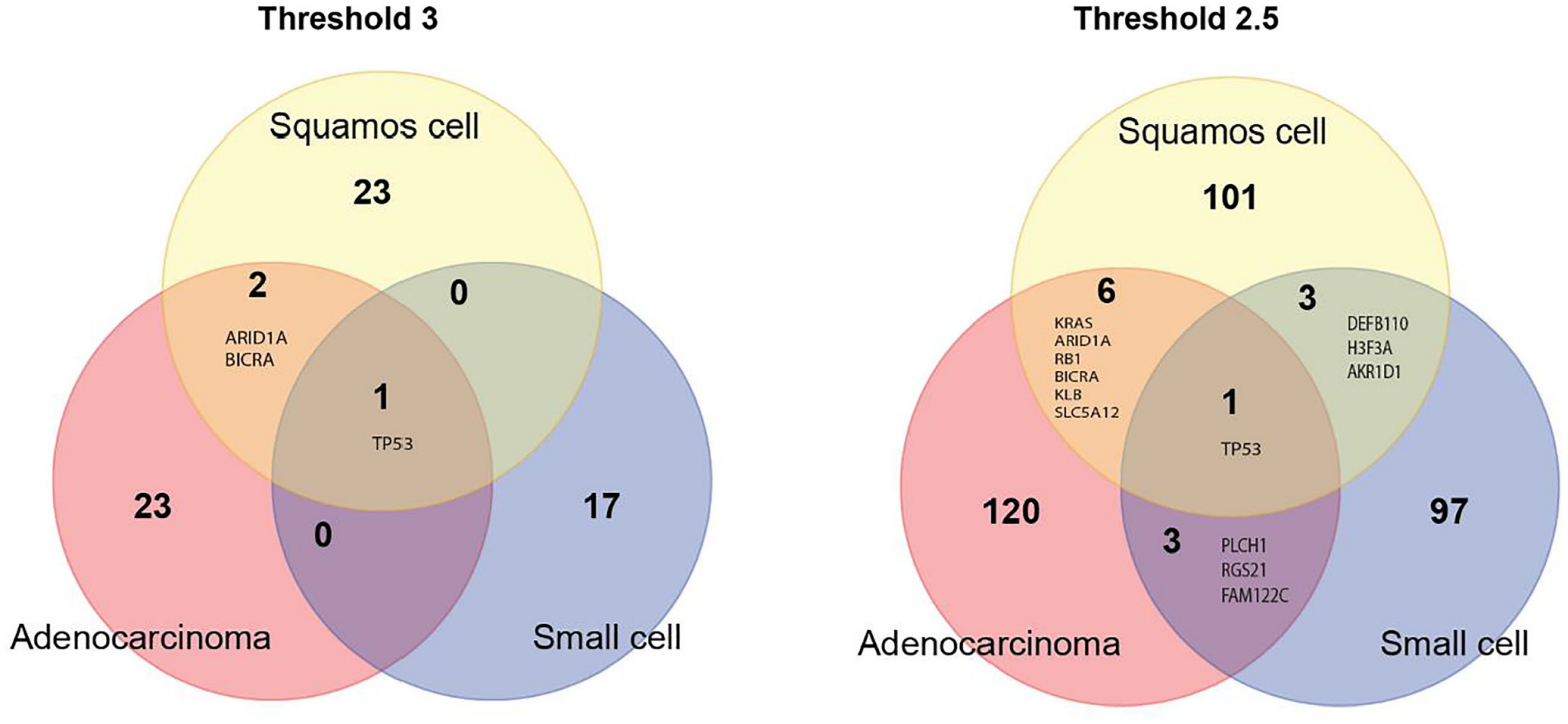
Venn diagram of candidate genes for the three major lung cancer cell types identified using the very strict threshold of 3 (left panel) and a more liberal threshold of 2.5 (right panel).

Three candidate genes, *ARID1A, BICRA* and *TP53*, were shared by adenocarcinoma and squamous cell carcinoma. Small cell lung cancer shared only *TP53* with adenocarcinoma and squamous cell carcinoma (Figure 4, left panel). When the threshold for candidate genes was lowered to 2.5, the number of shared genes increased to 7 for adenocarcinoma and squamous cell carcinoma, and to 4 between adenocarcinoma and small cell lung cancer and between squamous cell carcinoma and small cell lung cancer (Figure 4, right panel).

## DISCUSSION

There are two major novel elements in approach we have used in this study: (1) Using the exact number of potential sites for missense, silent and nonsense mutations in individual human transcripts determined by the *in silico* approach, instead of the size of transcript, a predictor typically used as a proxy for the number of mutation sites. (2) Using only silent mutations rather than all mutations together to estimate nucleotide context-dependent mutability to avoid biases related to selection of functional somatic mutations. These two elements improve the assessment of the expected number of somatic mutations which is critical for the identification of genes with an excess of potentially functional missense and nonsense mutations in them.

We found a two orders of magnitude variation in mutation rates across NCD-SNSs (see Supplementary Figure 1 and Supplementary Table 1). NCD-SNSs with highest mutation rates were G>T_CGG, G>T_CGT, G>T_GGG for adenocarcinoma, C>T_TCG, G>T_CGG, G>A_CGA for squamous cell carcinoma, and G>T_CGG, G>T_CGT, and G>T_CGC for small cell lung cancer. The results are consistent with reported mutation signatures for all histologies analyzed together [14–16].

Squamous cell carcinoma had the highest mutation rate followed by small cell lung cancer and adenocarcinoma. The pattern supports epidemiological conclusions that squamous and small cell lung cancers are more strongly associated with smoking, a well-known mutagenic factor, than adenocarcinoma [17, 18]. This indicates that differences in tobacco smoke exposure contribute to the differences in overall mutation rates among the three major LC cell types.

The observed number of missense and nonsense mutations per site per sample compared to that of silent mutations may be used as a global estimate of selection, a higher mutation density suggesting a positive selection and a lower one – a negative selection. The results of the analysis indicate that both missense and nonsense mutations are positively selected for at an overall similar level [19]. Positive selection for missense and nonsense mutations appears to be stronger in small cell lung cancer compared to adenocarcinoma and squamous cell carcinoma (Figure 1C).

Based on the observed large variation in mutation rates among NCD-SNSs we expected that accounting for the mutation rate might improve the prediction of the number of mutations based on the number of potential sites in the transcript. We found that indeed accounting for the mutation rates improves the estimates of the expected number of missense and nonsense mutations across the three major lung cancer cell types. The excess of potentially functional mutations in a gene indicates that they are positively selected for and that the gene is cancer-relevant. The approach we have used is easily applicable to other cancer types, provided that there are enough somatic mutation data generated by exome wide mutation detection methods.

We found seven genes, *TP53, KRAS, ARID1A, RB1,* *BICRA, KLB and SLC5A12* common for adenocarcinoma and squamous cell carcinoma. *TP53* is a known cancer-gene commonly mutated in many cancer types including lung cancer [20]. *KRAS* plays an important role in lung cancer and other types of cancer [21, 22]. AT-Rich Interaction Domain 1A – ARID1A gene is a member of the SWI/SNF family regulators of transcription by altering the chromatin structure. *ARID1A* is shown to have a tumor suppressor activity [23], which is consistent with our observation of an excess of nonsense mutations in it. BRD4 Interacting Chromatin Remodeling Complex Associated Protein (BICRA) is a component of SWI/SNF chromatin remodeling complex. So far there is no published evidence that this gene is associated with cancer risk or development. The retinoblastoma susceptibility gene (RB1) is a known tumor suppressor gene involved in lung cancer development [24, 25]. Klotho Beta-Like Protein – KLB gene plays a role in immune response (cytokine signaling) [26]. The literature on the role of KLD in lung cancer is rather limited, though a recent study [27] found that overexpression of KLB inhibits lung tumor growth *in vivo*. Solute Carrier Family 5 Member 12 - SLC5A12 gene plays a role as an ion and glucose transporter. There is no published evidence that the gene is involved in lung tumorigenesis. However, a recent study [28] found that SLC5A12 is a prognostic marker in head and neck squamous cell carcinoma.

Small cell lung cancer shows less overlap with both adenocarcinoma and squamous cell carcinoma which is consistent with the fact that small cell lung cancer overall differs from non-small lung cancer [29]. We found four genes shared by squamous cell carcinoma and small cell lung cancer: *TP53, DEFB110, H3F3A* and *AKR1D1*. Defensin Beta 110 – *DEFB110* gene plays a role in innate immune system [30]. The gene may be considered as a novel lung cancer gene candidate since there is no published evidence for its involvement in cancer. H3.3 Histone A – *H3F3A* gene encodes H3 histone. H3F3A has been shown to promote lung cancer cell migration [31]. Aldo-Keto Reductase Family 1 Member D1 - AKR1D1 gene is involved in synthesis of steroid hormones. At present there is no published evidence that the gene is involved in cancer development.

We found four genes shared by adenocarcinoma and small cell lung cancer: *TP53, PLCH1, RGS21* and *FAM122C*. Phospholipase C Eta 1 – *PLCH1* gene plays a key role in inositol synthesis. There is no evidence for the gene’s involvement in cancer. Regulator Of G Protein Signaling 21 – *RGS21* gene encodes structural components of G protein-coupled receptor complexes. Currently there is no published evidence that *RGS21* is involved in cancer development. PABIR Family Member 3 – *FAM122C* gene has serine/threonine phosphatase inhibitor activity. There is no published evidence that this gene is associated with cancer risk or development. Therefore, our analysis identified known and novel candidate cancer genes common for three major lung cancer cell types. The complete list of candidate genes for all the three histologies can be found in Supplementary Table 2.

We found that nucleotide context (adjacent nucleotides) strongly influence mutability. We noted a significant variation among the three major lung cancer histologies for the absolute majority of the nucleotide context dependent single nucleotide substitutions (Supplementary Figure 1). These findings emphasize the importance of the cancer and histology type-specific estimates of mutability for the prediction of the expected number of somatic mutations in transcripts.

### Limitations of the analysis

Our analysis does not directly include gene characteristic, for example gene expression level, associated with mutability as independent predictors of the number of somatic mutation in the gene.

## MATERIALS AND METHODS

### Data used

We used somatic mutations data from the Catalog Of Somatic Mutations In Cancer (COSMIC) database [32, 33]. Only verified mutations (those with confirmed somatic origin) were used. To ensure that all genes were targeted equally, only somatic mutations detected by whole genome/whole exome sequences were used in the analysis. The database was accessed September 15, 2021. Lung cancer somatic mutation data were available for 40,021 transcripts from 18,622 genes. Table 2 shows the number of missense, silent and nonsense mutations for each histology.

**Table 2:** Numbers of missense, silent and nonsense mutations per sample

Histology	Number of samples	Number of mutations per sample			
		Missense	Silent	Nonsense	All
Adenocarcinoma	902	236.74	74.68	19.63	331.04
Squamous cell carcinoma	723	326.54	117.48	26.04	470.07
Small cell lung cancer	210	379.45	85.70	29.32	494.46

The three major histological types of lung cancer, adenocarcinoma, squamous cell carcinoma and small cell carcinoma, were analyzed separately. For adenocarcinoma, data for 902 tumors from 17 studies were used. The corresponding numbers for squamous cell carcinoma were 723 tumors from 8 studies, and for small cell lung cancer, 210 tumors from 8 studies. The PubMed IDs of the published studies whose data were used in this study are shown in Supplementary Materials.

### Nucleotide context-dependent single nucleotide substitutions (NCD-SNSs)

It is known that the mutation rate of a given single nucleotide substitution (SNS) depends on the adjacent nucleotides [34, 35]. Therefore, we estimated mutation rates in a nucleotide context-dependent (NCD) way. In total, 64 trinucleotides are possible in the human genome. There are three possible substitutions for the core (middle) nucleotide, which gives the total number of possible NCD-SNSs 64 × 3 = 192. We analyzed these 192 NCD-SNS separately. All mutations were annotated for the sense strand.

### Estimating the numbers of potential sites for silent, missense and nonsense mutations generated by individual NCD-SNSs

Reference sequences were downloaded from the Consensus Coding Sequence Database [36]. To estimate the number of potential sites for each mutation type, we considered all possible trinucleotides in each transcript. For each trinucleotide we computationally mutated the core nucleotide into 3 possible SNSs by replacing it with a different nucleotide. SNS may produce missense, silent or nonsense mutations depending on the type of SNS, e.g. A>T, and the position within the trinucleotide. For each of all possible 192 NCD-SNSs we counted the numbers of possible SNSs producing missense, silent and nonsense mutations and used them as the numbers of potential sites for each transcript. Supplementary Figure 3 illustrates the approach of counting the number of potential sites for missense, silent and nonsense mutations for different NCD-SNSs.

### Estimation of mutation rates for NCD-SNSs

Mutation rates were computed as the ratio of the observed number of silent mutations to the corresponding number of potential sites across the whole genome, divided by the total number of analyzed samples. For 30 NCD-SNSs it is impossible to generate silent mutations, and the number of potential sites for silent mutations for such NCD-SNSs equals zero. The list of these NCD-SNSs is shown in Supplementary Text. We used missense mutations to estimate mutation rates for NCD-SNS with zero numbers of potential sites for silent mutations. To do this we applied a linear regression model. For 162 NCD-SNSs for which both missense and silent mutations are available, we built a linear regression model with the mutation frequency based on the analysis of silent mutations as the outcome and the mutation frequency based on the analysis of missense mutations as the predictor. We used the regression equation to predict mutation rates for the 30 NCD-SNSs unable to produce silent mutations.

### Analysis of the association between the number of potential sites and the observed number of likely functional missense and nonsense mutations

For each transcript, Pearson’s correlation coefficients (*ρ*) between the number of potential sites and the observed number of mutations were computed across 192 NCD-SNSs. This was done separately for missense and nonsense mutations. We also computed the correlations between the observed numbers of mutations and the number of sites for NCD-SNS weighted (multiplied) by the corresponding mutation rate. Supplementary Figure 4 shows how the correlations were computed. As the next step, we compared the distributions of *ρ* for the weighted and unweighted number of sites. The goal of the comparison was to test if accounting for the mutation rate improves the prediction of the observed number of mutations.

### The expected numbers of missense and nonsense mutations in a transcript

The expected number of somatic mutations was computed assuming that the chances to find missense and nonsense mutations are the same as chances for silent mutations. To compute the expected number of mutations in a transcript, the numbers of sites for missense and nonsense mutations were multiplied by the corresponding mutation rates. The sum of the products across the 192 NCD-SNSs gives the expected number of mutations in the transcript: $E=\sum_{i}^{192}\left(n_{i}\times m_{i}\right)$ ; where *n_i_* is the number of NCD-SNS sites and *m_i_* is the corresponding estimated mutation rate.

### Logarithms of the ratios of the observed to the expected number of mutations

Our hypothesis was that genes with the excess of missense and/or nonsense mutations are cancer-related. We used LOG of the ratio of the observed to the expected numbers of mutations as a measure of the excess of somatic mutations in transcript. To account for gene characteristics that may influence the mutation rate in a gene-specific manner, we adjusted the LOG ratios for missense and nonsense mutations by the LOG ratios for silent mutations. This was done by subtracting LOG ratios for silent mutations from the LOG ratios for missense and nonsense mutations. Our rationale for doing it was that if a gene has a high intrinsic propensity to mutate, this gene will have a high LOG ratio for silent mutations, so subtracting it from the LOG ratio for missense or nonsense mutations will provide an adjustment for the gene’s overall higher propensity to mutate.

## SUPPLEMENTARY MATERIALS






