# Supplementary material for: Identification of lung cancer drivers by comparison of the observed and the expected numbers of missense and nonsense mutations in individual human genes
Source: Oncotarget. 2022 May 25;13:756–67. doi: 10.18632/oncotarget.28231 (PMC9132259; doi:10.18632/oncotarget.28231)
Supplement: Supplementary file 1 [file oncotarget-13-28231-s001.pdf]

Identification of lung cancer drivers by comparison of the observed and the expected numbers of missense and nonsense mutations in individual human genes

SUPPLEMENTARY MATERIALS

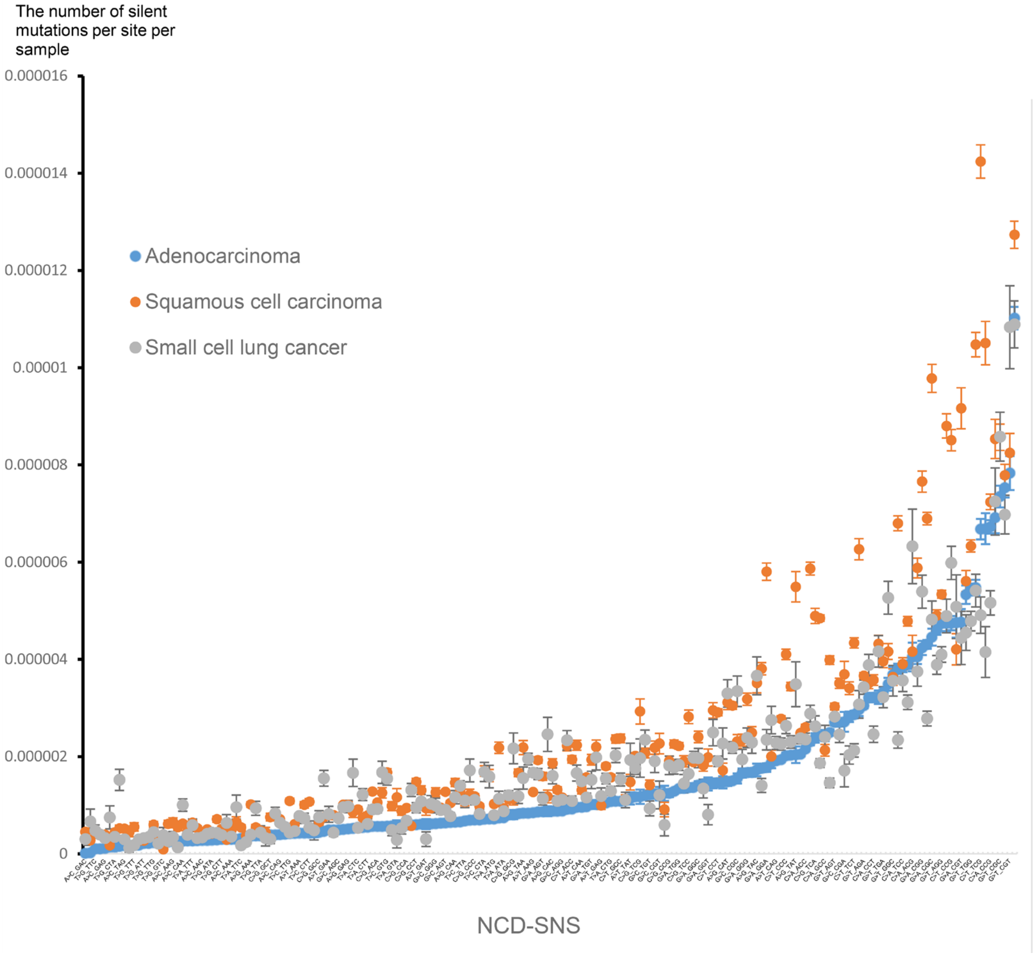

Supplementary Figure 1: Mutation rates (the number of silent mutation per site per sample) for 192 possible NCD-SNSs across 3 major lung cancer histologies, arranged from least to greatest in adenocarcinoma. Vertical bars are standard errors of mean.

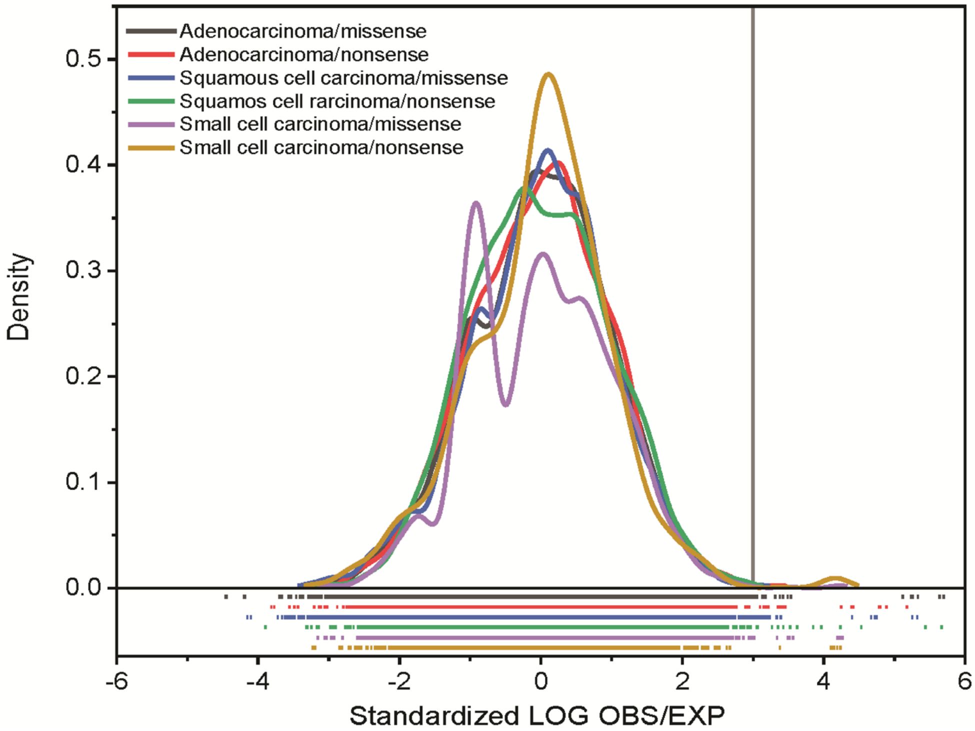

Supplementary Figure 2: Standardized distributions of the LOG(OBS/EXP)$_{mis\_or\_non}$ - LOG(OBS/EXP)$_{silent}$. The vertical line shows the threshold for the transcripts with an excess of missense or nonsense mutations in adenocarcinoma, squamous cell carcinoma and small cell lung cancer.

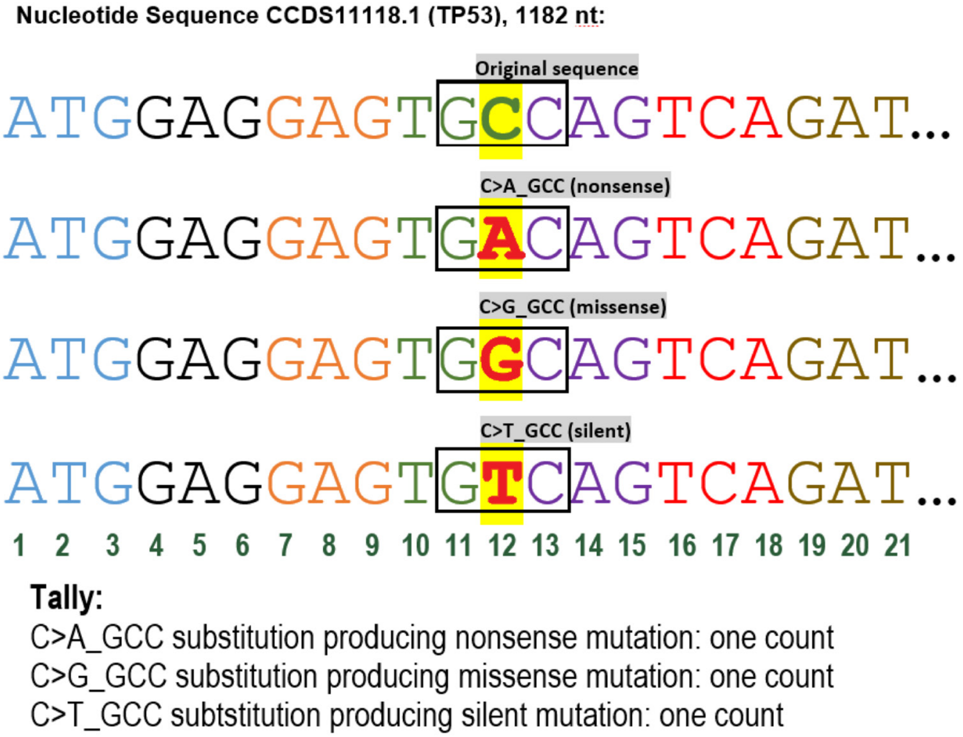

Supplementary Figure 3: Counting the number of potential sites for missense, silent and nonsense mutations. Shown is the beginning of the sequence for TP53 gene. Codons are shown by different colors. We computationally mutated position 12 corresponding to the "C" nucleotide in a trinucleotide GCC (marked by a frame). The core/central nucleotide "C" can generate 3 substitutions: C>A_GCC, C>G_GCC, and C>T_GCC. This core nucleotide is in the third position of the codon TGC; therefore, based on the codon table, the listed substitutions will generate, correspondingly, a missense, a nonsense, and a silent mutation. To count the total number of potential sites we go through all nucleotides across all transcripts in the human genome.

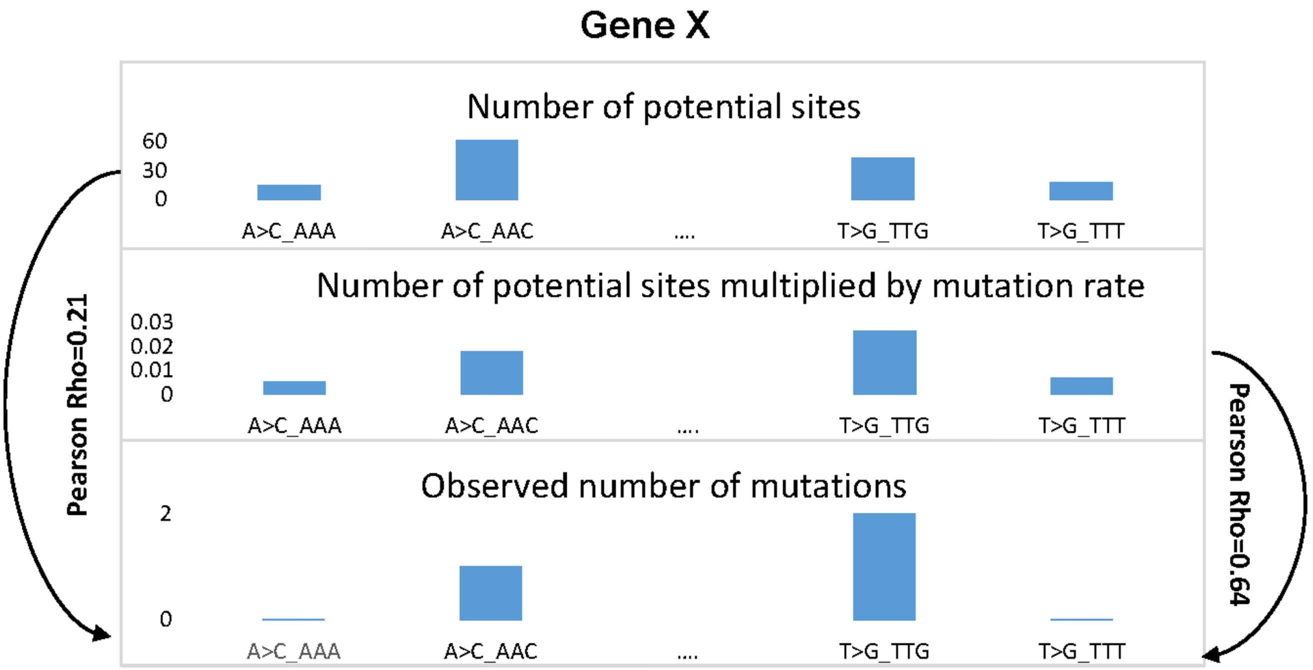

Supplementary Figure 4: A schematic representation of the computation of the correlations between the observed number of mutations and the number of the potential sites, both raw and weighted by the corresponding mutation rate.

Supplementary Table 1: Mutation rates estimated as the number of silent mutations per site per sample across 192 NCD-SNSs in 3 lung cancer histologies. See Supplementary Table 1.

Supplementary Table 2: The complete list of candidate genes for adenocarcinoma, squamous cell carcinoma, and small cell lung cancer predicted by an excess of missense or nonsense mutations. See Supplementary Table 2.

The list of NCD_SNS incapable to produce silent mutations

A>C_AAA, A>C_AAC, A>C_AAT, A>T_AAA, A>T_AAC, A>T_AAG, A>T_AAT, C>A_ACA, C>A_ACC, C>A_ACT, C>G_ACA, C>G_ACC, C>G_ACG, C>G_ACT, G>C_AGA, G>C_AGC, G>C_AGG, G>C_AGT, G>T_AGA, G>T_AGC, G>T_AGG, G>T_AGT, T>A_ATA, T>A_ATC, T>A_ATG, T>A_ATT, T>G_ATA, T>G_ATC, T>G_ATG, T>G_ATT

PubMed IDs of the papers whose data we used in the analysis

Lung adenocarcinoma

12068308, 22980975, 16140923, 23405175, 24449147, 25189529, 23733853, 22696596, 23856246, 26647728, 27900369, 22510280, 25112956, 27545006, 22975805, 29681454, 27923066

Squamous cell carcinoma

25112956, 23856246, 25189529, 23799614, 29681454, 22696596, 22510280, 26503331

Small cell lung cancer

12068308, 19330029, 20016488, 27873319, 22941188, 22941189, 30224629, 26168399
